# Toward mechanistic modeling and rational engineering of plant respiration

**DOI:** 10.1093/plphys/kiad054

**Published:** 2023-01-31

**Authors:** Philipp Wendering, Zoran Nikoloski

**Affiliations:** Systems Biology and Mathematical Modeling, Max Planck Institute of Molecular Plant Physiology, 14476 Potsdam, Germany; Bioinformatics, Institute of Biochemistry and Biology, University of Potsdam, 14476 Potsdam, Germany

## Abstract

Plant respiration not only provides energy to support all cellular processes, including biomass production, but also plays a major role in the global carbon cycle. Therefore, modulation of plant respiration can be used to both increase the plant yield and mitigate the effects of global climate change. Mechanistic modeling of plant respiration at sufficient biochemical detail can provide key insights for rational engineering of this process. Yet, despite its importance, plant respiration has attracted considerably less modeling effort in comparison to photosynthesis. In this update review, we highlight the advances made in modeling of plant respiration, emphasizing the gradual but important change from phenomenological to models based on first principles. We also provide a detailed account of the existing resources that can contribute to resolving the challenges in modeling plant respiration. These resources point at tangible improvements in the representation of cellular processes that contribute to CO_2_ evolution and consideration of kinetic properties of underlying enzymes to facilitate mechanistic modeling. The update review emphasizes the need to couple biochemical models of respiration with models of acclimation and adaptation of respiration for their effective usage in guiding breeding efforts and improving terrestrial biosphere models tailored to future climate scenarios.

## Introduction

Estimates indicate that plants release almost half of assimilated carbon dioxide (CO_2_) back into the atmosphere by the process of respiration and that this amount varies between species, conditions, and available resources (Ryan, 1991). The release of CO_2_ by plant respiration, relative to the net assimilation of CO_2_ by photosynthesis, determines the plant growth, carbon use efficiency (i.e., 1 − the ratio of net carbon gain to gross carbon assimilation in a given period), and turnover of carbon, and thus affects the global carbon cycle (Huntingford et al., 2017; Heskel, 2018). Since increases in atmospheric CO_2_ concentrations lead to warming of the planet, respiration also directly affects the climate. Therefore, understanding the factors that affect plant respiration is necessary for devising strategies to mitigate negative effects of future climate scenarios.

**Figure 1:**
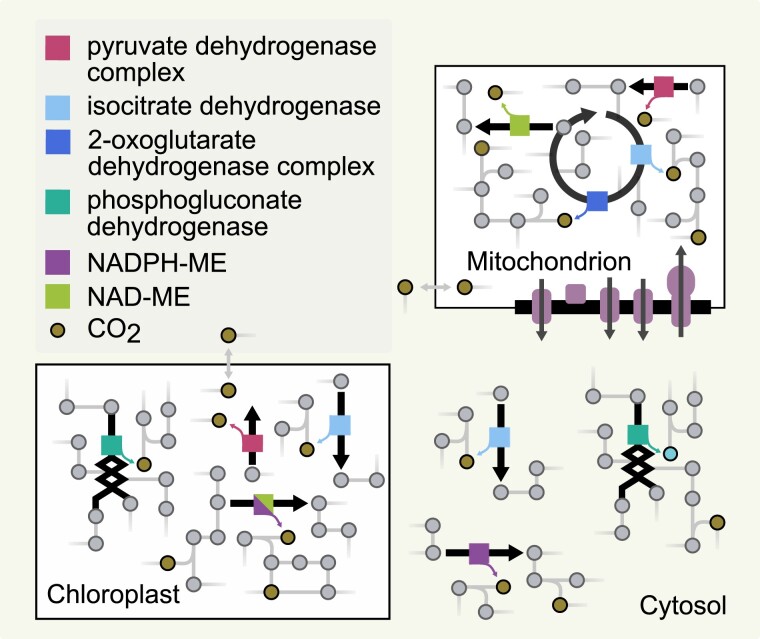
The contrast between the biochemical and physiological level of studying plant respiration. The figure illustrates the six enzymes considered in the biochemical view of plant respiration, distributed across three compartments (i.e. chloroplast, mitochondrion, cytosol), marked with different colored boxes (see legend). The physiological level considers all CO_2_ producing and consuming reactions, excluding photosynthesis and photorespiration—which are part of the entire metabolic network.

Respiration has been defined and studied at the biochemical and physiological levels (O’Leary et al., 2019). At the biochemical level, the respiration rate is characterized by the rate of oxygen (O_2_) consumption, as the terminal electron acceptor, or by the rate of CO_2_ produced by glycolysis, the oxidative pentose phosphate pathway (PPP), the tricarboxylic acid (TCA) cycle, and the mitochondrial electron transport chain (reviewed in O’Leary et al., 2019). More specifically, the biochemical definition of respiration considers CO_2_ released by (1) decarboxylation of pyruvate connecting glycolysis and the TCA cycle, (2) isocitrate dehydrogenase, and (3) 2-oxoglutarate dehydrogenase in the mitochondrial TCA cycle as well as during (4) the formation of ribulose 5-phosphate from glucose in the oxidative PPP operating in the cytosol and chloroplast and (5) decarboxylation of malate in the mitochondria by the NAD/NADP-dependent malic enzyme (Fig. 1). At the physiological level, of a plant organ or individual, the respiration rate is defined as the net gas (CO_2_ or O_2_) exchange, including processes beyond those considered at the biochemical level. In line with the effect that plant respiration has on the atmospheric CO_2_ concentration, Sweetlove et al., 2013 consolidated the definitions of respiration by focusing on net CO_2_ evolution, defined by the differences between all CO_2_-producing steps and all CO_2_-consuming steps, excluding photosynthesis and photorespiration (Fig. 1). This definition allows us to study respiratory CO_2_ release and diffusion across organelles, cells, tissues, or organs as well as spatially aggregated over larger scales (e.g. entire plant). Plant respiration serves three distinct purposes: (i) production of energy (ATP) and reducing power (NAD(*P*)H) from ADP and NAD(*P*), respectively, by consuming carbon sources generated by photosynthesis, (ii) production of carbon-rich precursors for biomass, and (iii) redox balancing (O’Leary et al., 2019). Respiration takes place both under day and night conditions to provide energy for all energy-requiring processes. During the day, plants also generate energy and reduce power by the light reactions of photosynthesis that also support energy-requiring processes. Therefore, the rate of day respiration is lower than the rate of night (nocturnal) respiration (Brooks and Farquhar, 1985; Cannell and Thornley, 2000; Tcherkez and Atkin, 2021). The change in plant metabolism due to light availability provides a clear distinction between modeling of respiration in autotrophic and heterotrophic organisms (e.g. yeasts, Elsemman et al., 2022).

Advances Box

- Kinetic and constraint-based modelings of plant central metabolism predict respiration rates that quantitatively agree with measurements over different environments.
- Maintenance costs, estimated using flux balancing and metabolic flux analysis, provide insights into the contribution of different pathways of central metabolism to CO_2_ release.
- Large-scale metabolic networks of model plants and crops show distinct patterns of day respiration and carbon use efficiency and pave the way for metabolic engineering of respiration rate.

Against this background, modeling can offer valuable tools to study plant respiration. Models of plant respiration, particularly at coarser scales, are predominantly phenomenological, based on an empirical relationship between plant respiration and other measured traits (often with a modest coefficient of determination of ∼0.6 (Reich et al., 2008)). For instance, based on the empirical support, it is often assumed that respiration is a constant factor of (i) photosynthesis (or gross carbon gain), allowing the usage of models of photosynthesis to predict respiration (Aber et al., 1996; Waring et al., 1998), and refer to “Steady-state and kinetic models relating respiration and growth” section (for a brief review, see Smith and Dukes, 2013); and (ii) organ-specific nitrogen content (Cox et al., 2000; Reich et al., 2008; Huntingford et al., 2017) or RuBisCO content, as the largest nitrogen investment made by a plant (De Pury and Farquhar, 1997). While these models capture moderately strong relationships present in the data, measured over some environments and species, the empirical relationships may not hold in others, raising issues with respect to the generalizability of the predictions. Further, phenomenological models of respiration do not distinguish between day and night respiration, despite the evidence against it at both the leaf (Heskel et al., 2013) and the ecosystem scale (Wehr et al., 2016). Phenomenological models are also at the core of predicting the effects of rising temperature on log-transformed measurements of plant respiration, with approaches based on fitting degree-two polynomials (Heskel et al., 2016) and modification thereof (Huntingford et al., 2017). These approaches establish a relation to acclimation of respiration based on the maximal velocity of RuBisCO carboxylation (Wang et al., 2020; Xu et al., 2021), and fitting parameters from the Arrhenius or the Eyring equation (Liang et al., 2018). Yet, phenomenological models are, intriguingly, at the core of all existing terrestrial biosphere models (Thornley and Cannell, 2000; Smith and Dukes, 2013; Fatichi et al., 2019).

Mechanistic models of respiration consider the rates of the reactions that contribute to net CO_2_ evolution, depending on the definition used. However, early attempts at mechanistic modeling of respiration have focused on characterizing the rate of each process (rather than individual reactions) that utilizes the energy produced by respiration along with the specific unit cost of the process, in terms of CO_2_ released or carbon source consumed (see Dewar, 2000 and Thornley and Cannell, 2000 for reviews). These attempts either estimated specific unit costs from experiments (e.g. carbon cost for growth) or calculated them (e.g. in terms of minimum ATP/NADPH needed) for uptake of ions (including nitrogen), nitrate reduction, symbiotic dinitrogen fixation, and phloem loading given simplified representation of the underlying biochemical processes. For other processes, including protein turnover, maintenance of cell ion concentrations and gradients, and futile cycles, estimates of specific costs are difficult to obtain, and it has been argued that they should be modeled phenomenologically (Thornley and Cannell, 2000). As a result, early mechanistic models of respiration do not consider a detailed representation of the underlying biochemical reactions. While they allow the study of the relation between respiration and whole plant growth models (at the cost of modifying phenomenological coefficients), they cannot be used to design rational strategies to engineer respiration via manipulation of rates of individual reactions.

To say that we have a good understanding of plant respiration is tantamount to demonstrating that we can: (i) accurately predict plant respiration and its plasticity to a given environmental cue for a given plant tissue/individual at a particular stage of development and (ii) devise rational manipulation strategies to modulate plant respiration, based either on genetic modifications (e.g. knock-downs/outs, overexpression, and combinations thereof) or breeding techniques. We argue that to address these problems, we need to develop mechanistic models based on first principles that include the biochemical details of the underlying reactions and allow for prediction/estimation of reaction fluxes (see Box 1 for an overview). Rates (or fluxes) of biochemical reactions are bounded by the turnover numbers and abundance of respective enzymes; reaction fluxes depend on and determine the concentration of metabolites used as substrates and products. Since enzyme abundances and metabolite concentrations differ between organs and developmental stages and are affected by the environment, the respiratory CO_2_ release is expected to differ between cellular contexts. Mechanistic modeling can also be very useful in identifying how the dependence on the rates of certain reactions and pathways leads to a certain empirical relationship detected across different species and conditions. Finally, mechanistic models of plant respiration can also be used to quantify the contribution of different cellular pathways to net CO_2_ evolution. In doing so, insights from mechanistic models of plant respiration can propel our understanding of the change in respiration over short time due to changes in the environment, referred to as acclimation (Atkin and Tjoelker, 2003), as well as the change in respiration over multiple generations exposed to persistent trend in an environmental cue (e.g. temperature and atmospheric CO_2_ concentration), referred to as adaptation (Smith and Dukes, 2013).

Box 1 Mathematical approaches for modeling respiration

The Box Fig. 1 shows the three types of approaches that have been used to model plant respiration: **A)** reaction–diffusion systems, **B)** kinetic models, and C) steady-state models. All modeling approaches can be mathematically described by a system of partial differential equations, given by the equation in the gray box. This equation describes the change in the concentration *x* over time $\partial_{t}x$ of modeled entities in terms of diffusion ($D\nabla^{2}x$) and effects of reactions/processes that shape concentration of these entities, R(x). The outcome of the equations is a simulation of spatiotemporal concentration profiles, shown by the contour plots in panel A. Without diffusion (D=0), the equations reduce to a system of ordinary differential equations (ODEs). An ODE can be used to simulate temporal concentration profiles (e.g. for two different entities, in blue and green, in **B**). In addition, if there is no change in concentration over time, the equations reduce to a system of polynomial equations (R(x)=0). Focusing only on the rates of the reactions/processes, the analysis can characterize all steady-state flux distributions that form the flux cone (top part, **C**). With additional constraints on growth and exchange fluxes, steady-state modeling can also determine the range of fluxes of the considered reactions/processes (bottom part, **C**). There are three variants of steady-state modeling, as shown in Box Fig. 1: **D)** classical constraint-based modeling, which results in a steady-state flux distribution (marked in red) that optimizes an objective (e.g. minimizing total flux); the steady-state flux distribution is one point in the flux cone (top part, **C**), E. enzyme-constrained modeling, which considers constraints due to available enzymes (blue squares) and their turnover numbers (k) and total protein mass (gray square); note that steady-state flux distribution that minimizes total enzyme usage may differ from the one that minimizes total flux (shown in **D**) and **F)** resource allocation modeling, which includes the translation and transcription machinery (ribosomes shown in green) and captures the synthesis (with dotted lines) and degradation of proteins. Models in **C–F** are suitable to study the contribution of different biochemical processes to respiration, including those that in classical mechanistic models are represented by phenomenological coefficients. Models in **A** and **B** can be explored on the biochemical and physiological level and are suitable to study acclimation of respiration.

**Box Figure 1:**
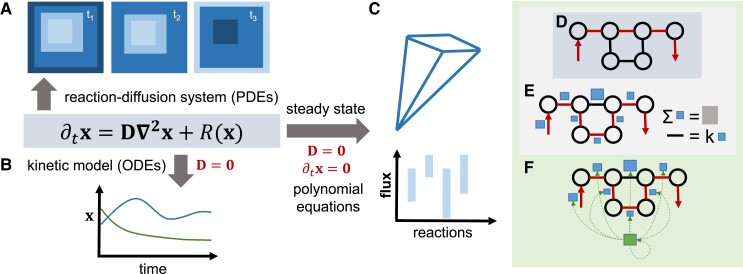
Overview of mechanistic approaches to model respiration.

The aim of this update review is to highlight the advances made in modeling plant respiration at the interface of the questions addressed by mechanistic modeling. We also provide a detailed account of the existing resources, with respect to reactions and kinetic properties of underlying enzymes that can further propel mechanistic modeling of plant respiration. In doing so, we provide a comparative overview of predictions of respiration from metabolic models of several photosynthetic eukaryotes following the constraint-based modeling framework. We further emphasize the need to couple biochemical models of respiration with models of acclimation and adaptation to guide crop breeding efforts for future climate scenarios.

### Classification of plant respiration models

The existing mechanistic models of plant respiration can be classified based on: (i) the mathematical formalism used, (ii) the level of biochemical details considered, and (iii) the spatiotemporal scales modeled. Based on the mathematical formalism, models of plant respiration can be grouped into reaction–diffusion, kinetic, and steady state (see Box 1). Reaction–diffusion models are employed to simulate and predict spatiotemporal distribution and diffusion of CO_2_ concentration at a subcellular, cell, and organ level. In contrast, kinetic models are used to simulate temporal trajectories (as well as steady states) of plant respiration rates and CO_2_ concentration. Mass balance modeling in turn can be used to predict the steady-state flux distributions over a range of environments—without the need to specify the dependence of fluxes on metabolite concentrations. Steady-state flux distributions can be predicted by constraint-based modeling that relies on optimizing a cellular objective (e.g. like growth in flux balance analysis [FBA] (Orth et al., 2010) assumed or shown to govern the functionality of plant cells and organs. Due to the dependence between the presented mathematical formalisms, hybrid models can also be created by using predictions of kinetic models to constrain steady-state models and vice versa.

The level of biochemical detail provides another axis to classify the models of plant respiration. For instance, biochemically detailed mechanistic models of respiration describe reaction rates by enzyme kinetic laws (e.g. mass action or Michaelis–Menten), capturing the dependence of the rates on metabolite (including carbon substrate for respiration) and enzyme concentrations. Mathematical descriptions of reactions rates usually involve numerous parameters that denote measurable properties of enzymes (e.g. turnover number, k_cat_) and the substrate affinity for an enzyme (e.g. Michaelis–Menten constant K_M_). Models at a coarser scale establish a relation between respiration and organ traits, like biomass, described by mathematical functions that do not pertain to molecular entities (i.e. enzymes and/or metabolites). From this elaboration, another implicit axis for model classification includes the spatiotemporal scales, with models at coarser scales tending to incorporate empirical/phenomenological relationships between the modeled quantities.

In the following subsections, we review the recent advances in modeling of plant respiration, while highlighting links to classical approaches used to model plant respiration. In addition, we critically assess the possibility to use these models to design strategies to engineer respiration.

### Steady-state and kinetic models relating respiration and growth

Early mechanistic models of respiration rate, *R* (mol CO_2_ time^−1^), aimed to bridge spatiotemporal scales by establishing a relation between respiration rate and plant growth over a specified period of time (often 24 h, for a comprehensive review, see (Amthor, 2000; Cannell and Thornley, 2000; Thornley and Cannell, 2000)). Two paradigms have been predominant in this line of modeling: the growth-and-maintenance-respiration paradigm and the general paradigm.

Following the growth-and-maintenance-respiration paradigm, the respiration rate can be described as follows:


(1) $$R=R_{G}+R_{M}=g_{R}G+m_{R}W=g_{R}\frac{dW}{dt}+m_{R}W,$$

with *R*_*G*_ denoting the growth respiration, comprising all processes that contribute to plant growth, *G* (mol C time^−1^), and *R*_*M*_, the maintenance respiration, that includes all processes not directly related to the accumulation of biomass, *W* (mol C) (see “Introduction” section). Here, *g*_*R*_ (mol CO_2_ mol C^−1^) denotes the growth respiration coefficient and *m*_*R*_ (mol CO_2_ mol C^−1^ time^−1^) denotes the maintenance respiration coefficient (Amthor, 2000). In addition, *m*_*R*_ can be estimated as $\underset{r}{\sum }\;\alpha_{r}v_{r}$, with *α*_*r*_ denoting the specific unit costs (in CO_2_ units) of reaction/process *r* and *v*_*r*_ denotes the specific rate of *r* (CO_2_ mol mol C^−1^ time^−1^). Since growth and maintenance processes are difficult to disentangle (Amthor et al., 2019), and in light of the decomposition of *m*_*R*_ , the respiration rate, *R*, can be cast, like *m*_*R*_ mentioned earlier, as $\underset{r}{\sum }\;\alpha_{r}v_{r}$ following the general paradigm (Amthor, 2000).

To calculate *g*_*R*_, one uses knowledge of pathway biochemistry, substrates used, and biomass composition generated by the activity of the growth-related pathways, thus providing a link between the biochemically detailed models of metabolism and the coarser paradigms for modeling respiration. This approach is based on calculating the required ATP and NAD(P)H needed to produce the biomass composition components and determining carbon substrate required to match these energy demands (Penning De Vries et al., 1974). As a result, *g*_*R*_ and *m*_*R*_ are not fixed but depend on the biomass composition and the activity of reactions that change with the developmental stage and environmental cues. Since plant metabolic networks are context specific (e.g. different tissues, day/night scenarios) (Robaina Estévez and Nikoloski, 2015) and plant tissues have different biomass composition (Seaver et al., 2015), the specific unit costs are expected to differ between tissues. This is due to the differential rates of reactions between contexts; for instance, marked differences in rates of reactions, considered in the biochemical definition of respiration, can be observed between day and night scenarios in the case of Arabidopsis (*Arabidopsis thaliana*) (Supplemental Fig. S1). The specific unit costs can be automated by using constraint-based modeling of plant metabolism (Arnold and Nikoloski, 2014; Arnold et al., 2015) (see Box 1). For instance, ATP costs for the synthesis of all amino acids were determined in three metabolic models of Arabidopsis, showing that the costs highly depend on the model used and the simulated trophic growth, with differences as high as 2-fold (Arnold et al., 2015). Yet, these approaches have not been used in the growth-and-maintenance-respiration paradigm. While the formulation of Eq. (1) allows us to directly investigate the effect of altering *g*_*R*_ and *m*_*R*_ on respiration, the mechanistic details of how these alterations can be carried out in practice (e.g. gene knock-out) and their effect on metabolism are challenging to pinpoint following this paradigm.

With the assumption that carbon loss other than in the form of CO_2_ is minor (that often does not hold due to exudates from roots or leaves (Dong et al., 2021)), $R\approx P-\frac{dW}{dt}$, with *P* denoting the photosynthesis rate (mol CO_2_ time^−1^). As a result, one obtains that $G=\frac{dW}{dt}=\frac{1}{1+g_{R}}(P-m_{R}W)$ (Amthor et al., 2019), where $\frac{1}{1+g_{R}}$ denotes the yield of growth processes (i.e. amount of growth per unit substrate used in growth processes) (Thornley, 1970). Using the plant growth model by Ågren (Ågren, 1996), whereby the total nitrogen can be divided into nitrogen in proteins used for growth and structurally bound nitrogen (with carbon, *C*, after subtraction of respiratory losses), *N* = *N*_*p*_ + *γC*, it follows that $\frac{dC}{dt}=\phi_{CN}N_{p}$ represents the net photosynthesis rate, with *ϕ*_*CN*_ denoting the nitrogen to carbon conversion factor. Assuming the balanced plant growth (Ågren, 2004) and that the concentration of carbon is proportional to *W*, i.e. *W* = *kC*, it follows that $\frac{dW}{dt}=\frac{1}{1+g_{R}}\left(\frac{\phi_{CN}N_{p}}{k}-m_{R}W\right)=\frac{1}{1+g_{R}}\left(\frac{\phi_{CN}N_{p}}{k}-\phi_{CN}\gamma W-m_{R}W\right)$ (Chen et al., 2022). This extension can be used to predict the ratio of *N* and *C* in plant biomass based on photosynthesis, maintenance respiration, and relative growth rate. This model has been related to the phenomenological allometric law describing the relationship between total *N* and biomass, *W*, yielding the empirical Richards growth model (Richards, 1959). Growth respiration has also been included in a model of seedling growth (parameterized for tomato (*Solanum lycopersicum*)) as proportional to the structural growth rate; the model aims to also predict the diurnal changes in nonstructural carbohydrates by employing phenomenological models for the considered processes (Gent, 2018). It is based on the earlier efforts to integrate the growth-and-maintenance-respiration paradigm in models for the dynamics of nonstructural carbohydrates and plant growth (Gent and Enoch, 1983; Gent and Seginer, 2012).

The growth-maintenance-respiration paradigm is also at the core of a biophysical model for tree stem respiration (TReSpire) (Salomón et al., 2020). The model couples water and carbon fluxes in tree stems by combining three submodels for (i) water flow and storage, (ii) CO_2_ sources and fluxes, and (iii) stem carbon balance. As a part of the submodel for CO_2_ sources and fluxes, the maintenance respiration of xylem and outer tissues is modeled phenomenologically, as a function of nitrogen concentration (Westerband et al., 2022). The growth respiration in the cambium is a fixed proportion of growth, specified by the yield of growth processes. The model allows that the respired CO_2_ can be radially released into the atmosphere, dissolved in the sap solution, transported axially through the xylem, or stored within the stem in gaseous and liquid phases. The CO_2_ source and balance submodel is calibrated with measured stem CO_2_ efflux, while that of stem carbon balance relies on measured stem diameter and nonstructural carbohydrates in the stem. The model was used to compare diurnal stem respiration in cedar (*Thuja occidentalis*), Norway maple (*Acer platanoides*), and pedunculated oak (*Quercus robur*) trees (Salomón et al., 2022), and predicted that most (>87%) of the respired CO_2_ radially diffused to the atmosphere, while the remainder was transported upward with the transpiration stream (Salomón et al., 2022). A similar approach, but at a coarser level, was used to model forest growth (Collalti et al., 2016; Collalti et al., 2020) by establishing differential equations for the growth of live woody and green biomass based on turnover of the respective biomass parts (the key model parameter) and their production from carbon involved in structural components.

### Day respiration in steady-state models of photosynthesis

Day respiration, *R*_*d*_, defined as the net CO_2_ evolution association to nonphotorespiratory processes, is already embedded in the classical Farquhar–von Caemmerer–Berry (FvCB) model of steady-state photosynthetic assimilation (Farquhar et al., 1980), whereby the net rate of CO_2_ assimilation, *A*, at the level of a leaf is given by


(2) $$A=V_{c}-0.5V_{o}-R_{d},$$

where *V*_*c*_ is the rate of RuBisCO carboxylation and *V*_*o*_ is the rate of RuBisCO oxygenation. In contrast to respiration, the definition of gross rate of CO_2_ fixed by photosynthesis, given by *V*_*c*_ − 0.5*V*_*o*_ in Eq. (2), extends from the level of a single chloroplast to a cell and entire leaf (Farquhar et al., 1980). The original FvCB model and extensions thereof (Farquhar et al., 2001) integrate mechanistic insights by considering the limitation of *V*_*c*_ due to RuBisCO, ribulose-1,5-bisphosphate (RuBP) regeneration, and triose phosphate utilization, thus providing a mechanistic steady-state model of photosynthesis (Yin et al., 2021) that can predict net CO_2_ evolution and parameters derived from it (e.g. quantum yield of photosynthesis). Scaling this model to vegetation level assumes that respiration is 5.8% of the net rate of CO_2_ assimilation (Farquhar et al., 1980), irrespective of environments simulated. Further, a model of CO_2_ diffusion, based on the same reactions included in the FvCB model in Eq. (2), considers day respiration as a constant (0.8 µmol m^−2^ s^−1^) (Tholen and Zhu, 2011) and hence does not provide a mechanistic description of respiration. Another coarser model for CO_2_ diffusion provides a phenomenological description of day respiration (and the maximal carboxylation rate of RuBisCO) by fitting to the response curve of net CO_2_ assimilation to light intensity (irradiance) (Berghuijs et al., 2017). Nevertheless, this model can provide the means to estimate day respiration from gas-exchange measurements as well as re-assimilated CO_2_ due to (photo)respiration, as illustrated in a study with tomato cultivars (Berghuijs et al., 2019). The latter study demonstrated the dependence of the estimates of day respiration on re-assimilation of released CO_2_. These examples illustrate that mechanistic understanding of the net rate of CO_2_ assimilation—deemed fully resolved (Sweetlove et al., 2013) and integrated in vegetation, earth system, and climate models (Smith and Dukes, 2013)—cannot be achieved without having mechanistic insights in processes that affect plant respiration across the different scales.

A hybrid model for day respiration based on the general paradigm was developed by combining flux balance equations for ATP, NADH, and chloroplastic and cytosolic NADPH using the predictions from the FvCB model. These equations were solved for the nonphotorespiratory CO_2_ (Buckley and Adams, 2011), expressed as the sum of four rates: the chloroplastic and cytosolic oxidative PPP, the catabolic substrate oxidation, and the by-product of anabolic carbon flow (specified as input). Different scenarios were investigated in terms of nitrogen supply (nitrate vs. ammonium) and two leaf maturity levels (with high and low relative growth rates and different amino acid export capacity) by making use of the leaf composition data. This model could qualitatively reproduce experimental observations; for instance, respiration was the largest in the dark for young leaves supplied with nitrate, which needs more energy for assimilation. Nevertheless, the model is in part phenomenological since the maximum RuBP carboxylation and the maximum electron transport rates are assumed to be proportional to the leaf nitrogen content.

A labeling study with the supply of ^13^CO_2_ to the model plant camelina (*Camelina sativa*) found that less than 10% of day respiration results from the reactions in the TCA cycle reactions, and that oxidation of glucose-6-phosphate to pentose phosphate via 6-phosphogluconate in the oxydative PPP accounted for more than 93% of CO_2_ released by day respiration. In contrast to this modeling study, position-specific decarboxylation measurements in a labeling experiment with ^13^C-enriched glucose at specific C-atom positions supplied to sunflower (*Helianthus annus*) leaves found that decarboxylation rates increased, but not abruptly, at low light. This increase was largely due to the prevalence of pyruvate dehydrogenase (PDH) flux relative to that of the TCA cycle or the PPP (Gauthier et al., 2020). The differences in the findings may be due to the consideration of pools that are not at a steady state but were included in the modeling equations.

A recent study by (Bender et al., 2022) provided estimates for the rate of (net) nonphotorespiratory, uncompensated (NU) decarboxylation that corresponds to the decarboxylation rate associated with biosynthetic pathways when the TCA cycle is fully open. To this end, the study makes use of mass balancing in simplified models of algae and cyanobacteria to assess the contribution of decarboxylations that are made for the synthesis of carbohydrate, lipids, and amino acids (proteins). The conclusions drawn are that three sets of reactions are responsible for NU decarboxylation: (1) decarboxylation of pyruvate to make acetyl Co-A, used in the formation of citrate or lengthening of amino acid chains, (2) decarboxylation via isocitrate dehydrogenase to make α-ketoglutarate, and (3) decarboxylations associated with the synthesis of amino acids. The study found that the ratio of NU decarboxylation to net carbon assimilation ranges from 0.24 to 0.32, higher than the value of 0.22 obtained from flux-balanced analyses and fluxes estimated by fitting data from labeling experiments with models of algae and cyanobacteria. This ratio is ∼5-fold larger in comparison to land plants (Embryophytes), since during the day they invest more in the production of less costly carbohydrates than in lipids and proteins.

### Kinetic and hybrid models of plant respiration

Building on an earlier comprehensive model of photosynthesis and related pathways (Zhu et al., 2007), a kinetic model of plant primary metabolism, including photosynthesis, glycolysis, the TCA cycle, and nitrogen assimilation, was recently developed (Zhao et al., 2021). This kinetic model was used to investigate the steady-state photosynthetic net CO_2_ assimilation, photorespiration, day respiration, and nitrogen assimilation under different light and CO_2_ levels. The day respiration rate was modeled as the sum of CO_2_-producing reactions in the model. Most reaction rates are modeled according to the Michaelis–Menten law (with consideration of activation and inhibition, where known), with some of the parameters obtained from the literature. The missing parameters were estimated by fitting data only for the response of net CO_2_ assimilation to CO_2_ concentrations. As a result, the obtained predictions do not match the measured steady-state metabolite levels at different CO_2_ concentrations, particularly for CO_2_ levels above 100 ppm. Predictions from the kinetic model showed that day respiration at ambient conditions is in the range of measured values, that it decreases with increasing irradiance, and that the decrease is fast below 200 μmoL m^−2^ s^−1^, but slows down at higher irradiances. The model also predicted that day respiration decreases with the increasing internal CO_2_ concentration.

Stoichiometric models of primary metabolism of Arabidopsis have already been used to predict flux distributions under heterotrophic growth, with sucrose as the main respiratory and growth substrate, and found reactions involved in respiration (Fig. 1) to carry higher flux in comparison to photoautotrophic growth (de Oliveira Dal’Molin et al., 2010). Building on this and follow-up modeling efforts, a hybrid modeling approach, combining a stoichiometric model of plant central metabolism (Shameer et al., 2019) with a kinetic model of photosynthesis (Zhu et al., 2013), was recently proposed to make predictions of elevated CO_2_ concentrations on photosynthesis and night respiration for field-grown soybean (*Glycine max*) plants on a daily basis (Shameer et al., 2022). In the loose coupling version of the models, the predictions from the kinetic model about photosystem II flux and the ratio of carboxylation and oxygenation fluxes of RuBisCO were used as constraints in the flux balance analysis. In the tightly coupled version, the day stoichiometric model used the exchange from the chloroplast in the kinetic model as inputs to predict the rest of the fluxes using the flux balance analysis, allowing for some metabolites to accumulate. To this end, phenomenological relationships were imposed for the accumulation fluxes of malate, starch, and citrate, and the models were iteratively solved by checking for identical predictions of fluxes for the sink reactions of ATP and NADPH (included to account for any additional costs not considered in the kinetic model). The stoichiometric part of the hybrid model predicted that the oxydative PPP and PDH contributed a large fraction (∼30% each) of the night CO_2_ evolution, with a smaller proportion (∼10%) of the total CO_2_ respired due to the TCA cycle. The remaining minor contributors were due to the malic enzyme, allantoin degradation, and amino acid biosynthesis. In addition, a higher respiration rate was found under elevated CO_2_ (from 372 to 552 ppm), due to the higher transitory starch stored that is predicted by the kinetic component of the hybrid model.

In addition, a stoichiometric model including pathways involved in respiration was developed for tomato fruit pericarp (Colombié et al., 2017). The model was used in multi-phase flux balance analysis in which exchange fluxes were parametrized based on measured metabolite contents (Colombié et al., 2015). The model predicted the expected sharp peak at 40 days postanthesis for fluxes involved in energy metabolism and CO_2_ release from decarboxylation. This prediction is in line with the observation that the initiation of fruit ripening is often associated with a marked increase in respiratory CO_2_ evolution, termed the respiratory climacteric (Seymour, 1993).

### Challenges in modeling plant respiration

Since mechanistic biochemical models of plant respiration are based on specifying the rates of reactions that contribute to CO_2_ production and consumption, here we provide a succinct overview of reactions that contribute to CO_2_ evolution in large-scale models of plant metabolism. The overview highlights three challenges in modeling plant respiration: (1) diversity of enzymes and multicompartmentality of the processes involved, (2) uncertainty associated with estimates of enzyme kinetic properties, and (3) context specificities of the processes contributing to CO_2_ evolution.

### Diversity of enzymes and multicompartmentality of processes

**Figure 2:**
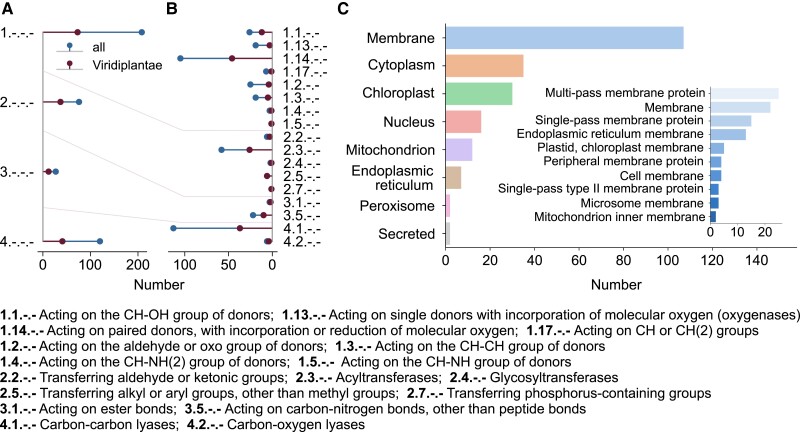
Functional classification of CO_2_-producing reaction and subcellular localization of corresponding enzymes. The distributions of EC numbers on the first A and second level B are shown for the entire set of CO_2_-producing reactions obtained from BioCyc (Karp et al., 2019) and BRENDA (Chang et al., 2021) (“all”) and green plants (Viridiplantae). C, CO_2_-producing reactions were obtained from BioCyc and BRENDA, and the associated EC numbers were used to query UniProt (Bateman et al., 2021) to retrieve subcellular localizations. Multiple locations of the same enzymes were considered, and the individual localizations were grouped into broader categories by using the following keywords: “Cytoplasm,” “Mitochondrion,” “Endoplasmic reticulum lumen,” “Endoplasmic reticulum membrane,” “Nucleus,” “Peroxisome,” “Secreted,” and “Chloroplast.” Finally, only localizations with more than one occurrence were used for the generation of this figure. The subdivision of the “Membrane” term into the original UniProt localization terms is shown in the inlay.

We used BioCyc (Karp et al., 2019) as a database of biochemical reactions and inspected the Enzyme Commission (EC) numbers (IUMBM, 1992) of the enzymes reported to catalyze CO_2_-producing reactions in green plants (Viridiplantae) and in the remaining organisms included in the database. Two additional EC numbers were added from CO_2_-producing reactions in BRENDA (Chang et al., 2021). We found that the largest number of enzymes in Viridiplantae that contribute to CO_2_ production include oxidoreductases (208), transferases (76), and lyases (120) (Fig. 2A). A more refined classification pointed out that enzymes acting on paired donors, with incorporation or reduction of molecular oxygen (EC 1.14.-.-), are predominant in the oxidoreductases, while acyltransferases (EC 2.13.-.-) and carbon-carbon lyases (EC 4.1.-.-) contribute most to the transferases and lyases, respectively (Fig. 2B). Considering the cellular compartments, we found that the largest proportion of the Viridiplantae CO_2_-producing enzymes is localized to the membrane (49.8%, with multi- and single-pass membrane protein having the largest contribution), then cytosol (16.3%), and chloroplast (14.0%), followed by the nucleus (7.4%) and mitochondrion (5.6%) (Fig. 2C). Protein localizations were obtained from UniProt (Bateman et al., 2021) using the set of aforementioned EC numbers as queries. As pointed by the findings shown in Fig. 2, the sheer number of compartments involved along with the enzymes/reactions considered represents the first challenge for creating detailed biochemical models to simulate and predict plant respiration rates over time and space.

### Uncertainty associated with estimates of enzyme kinetic properties

**Figure 3:**
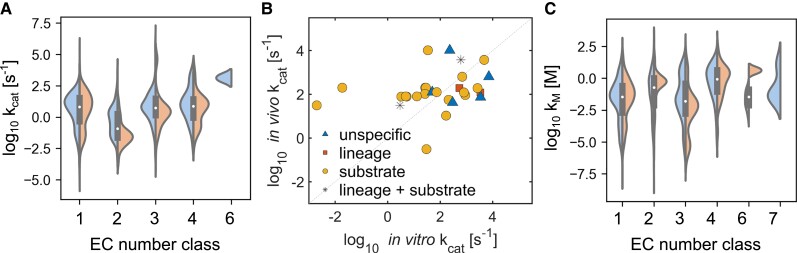
Challenges in developing kinetic models of respiration due to poor parameterization. In vitro measurements on turnover numbers (*k*_cat_) and Michaelis–Menten constants (K_M_) were obtained from BRENDA (Chang et al., 2021). A and C, The distributions of turnover numbers and Michaelis–Menten constants, respectively, within Viridiplantae (green plants) and all remaining taxa. B, The correspondence between in vitro measurements and maximal in vivo *k*_cat_ estimates for *Arabidopsis thaliana* (Küken et al., 2020) associated with glycolysis, TCA cycle, PPP, and oxidative phosphorylation. The in vitro turnover numbers from BRENDA were matched to EC numbers and reaction substrates in the AraCore model (Arnold and Nikoloski, 2014). The different confidence levels are listed in the increasing order of confidence: “unspecific” (only EC number matched), “lineage” (source organism shares a large part of its lineage with *A. thaliana*, but no substrate was matched), “substrate” (only (one) substrate match), and “lineage + substrate” (both substrate and lineage match). The dashed line indicates a perfect correlation of 1.

Estimates and measurements of turnover numbers or parameters that depend on turnover numbers (e.g. maximal enzyme velocity, V_max_) are used in the specification of the reaction rates in kinetic models. Turnover numbers are also used in protein-constrained models (Nilsson et al., 2017; Kerkhoven, 2022) and resource allocation models (Yang et al., 2018) of whole-cell metabolism, to date generated only for prokaryotes (see Box 1). The k_cat_ and V_max_ values used in these models are measured in vitro and often from different organisms, raising questions about the relevance of predictions obtained from such models (particularly if additional parameter fitting is involved). For instance, inspection of the distribution of k_cat_'s for the enzymes in Fig. 3A indicated differences between values obtained from Viridiplantae and remaining organisms; this was particularly the case for transferases (EC 2.-.-.-) and hydrolases (EC 3.-.-.-) that showed the tendency for smaller values in Viridiplantae (Fig. 3A). This observation would imply that bigger protein investment is needed to reach the same maximal velocity in organisms from Viridiplantae than organisms from other taxa. Interestingly, we could not identify measured k_cat_ values for ligases (EC 6.-.-.-) (Fig. 3A).

Further, the in vitro measurements of k_cat_ values in Arabidopsis have been shown not to correspond to in vivo estimates obtained by integrating fluxomics and quantitative proteomics measurements (Küken et al., 2020). By focusing on the enzymes involved in CO_2_ production from the pathways in Fig. 1 included in a metabolic model of Arabidopsis metabolism (Arnold and Nikoloski, 2014), we found that the in vitro measured k_cat_ values are often several orders of magnitude different in comparison to the in vivo k_cat_ estimates (Fig. 3B). Interestingly, like for k_cat_, we observed differences in the distributions of K_M_ values for the substrates of the enzymes that catalyze CO_2_-producing reactions (Fig. 3C). Therefore, obtaining reliable estimates of enzyme kinetic parameters represents the second challenge in creating mechanistic models of plant respiration that rely on these parameters to specify the enzyme kinetics that describes the dependence of reaction rates on metabolite concentrations.

### Context specificity of processes shaping plant respiration

**Figure 4:**
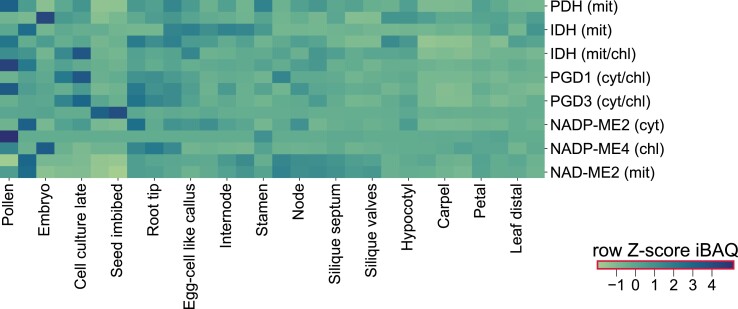
Challenge in developing context-specific metabolic models to study respiration. Protein abundances across 30 tissues were obtained for the enzymes described in Figure 1 (Mergner et al., 2020). The raw intensity based absolute quantification (iBAQ) values across the different tissues were Z-transformed for each enzyme separately. For enzyme complexes, the minimum value of the subunit expression values is shown. iBAQ values describe protein abundance, and they are obtained by dividing the sum of protein intensities by the number of possible tryptic peptides of a protein. PDH: pyruvate dehydrogenase, IDH: isocitrate dehydrogenase, 2OGDH: 2-oxoglutarate dehydrogenase, PGD: phosphogluconate dehydrogenase, ME: malic enzyme.

Even with complete knowledge of the kinetic properties of enzymes and reactions catalyzed by these enzymes, the activity of a reaction depends on the abundance of enzymes that varies between cellular contexts, such as tissue, organ, and developmental stages (Robaina Estévez and Nikoloski, 2014). As a result, differences in respiration rates are expected between different cellular contexts. By using a comprehensive proteomics dataset of Arabidopsis composed of 30 tissues (Mergner et al., 2020), we surveyed the abundances of CO_2_-releasing enzymes in the PPP, glycolysis, and TCA cycle as well as NAD- and NADPH-dependent malic enzymes depicted in Fig. 1. The columns in the heatmap shown in Fig. 4 are clustered based on the protein abundance across different tissues. We observed that enzymes catalyzing the same reaction show different abundance in both different subcellular compartments as well as different tissues or developmental contexts. For instance, all considered enzymes, except for NADP-ME1, exhibit their lowest abundance in seed and imbibed seed. Moreover, samples from cell culture show the highest expression of isocitrate dehydrogenase and phosphogluconate dehydrogenase. Further, root tissues show overall higher activity in TCA cycle and PPP enzymes, and NADP-ME4, indicating overall higher potential for CO_2_ release. Interestingly, the majority of the highest abundance of each CO_2_-releasing enzyme can be found in pollen and senescing leaf. These observations point to challenges related to the prediction of plant respiration due to the need to consider tissue-specific protein abundances and gene expressions (Supplemental Fig. S2) underlying the involved reactions and pathways.

### Perspective on modeling plant respiration

**Figure 5:**
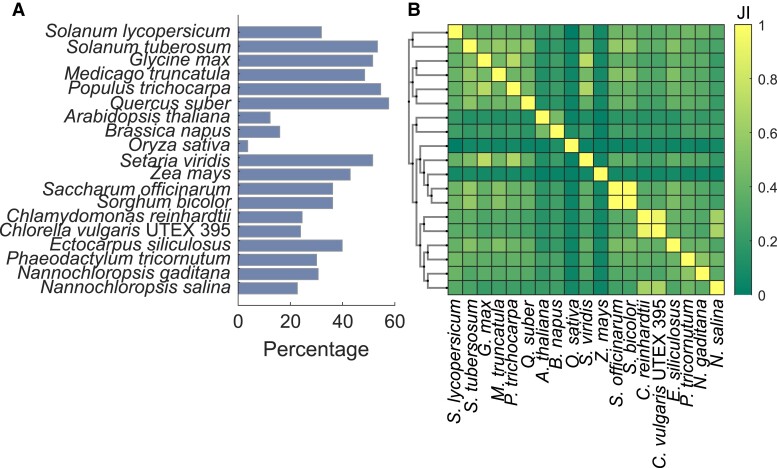
Challenge with complexity of processes that contribute to respiratory CO_2_ release. A, The set of EC numbers associated with CO_2_-producing reactions in BioCyc (Karp et al., 2019) and BRENDA (Chang et al., 2021) was compared to the EC numbers in genome-scale metabolic models of *Chlorella vulgaris* UTEX 395 (Zuñiga et al., 2016), *Setaria viridis* (Shaw and Cheung, 2019), *Nannochloropsis gaditana* (Shah et al., 2017), *Populus trichocarpa* (Sarkar and Maranas, 2020), *Ectocarpus siliculosus* (Prigent et al., 2014), *Medicago truncatula* (Pfau et al., 2018), *Glycine max* (Moreira et al., 2019), *Nannochloropsis salina* (Loira et al., 2017), *Phaeodactylum tricornutum* (Levering et al., 2016), *Chlamydomonas reinhardtii* (Imam et al., 2015), *Brassica napus* (Hay et al., 2014), *Solanum lycopersicum* (Gerlin et al., 2022), *Zea mays* (Simons et al., 2014), *Sorghum bicolor* (de Oliveira Dal’Molin et al., 2010), *Saccharum officinarum* (de Oliveira Dal’Molin et al., 2010), *Quercus suber* (Cunha et al., 2022), *Oryza sativa indica* (Chatterjee et al., 2017), *Solanum tuberosum* (Botero et al., 2018), and *Arabidopsis thaliana* (Arnold and Nikoloski, 2014). If not contained in the model, the sets of EC numbers were derived by pattern matching of reaction ID, names or notes, or ID matching of reaction IDs to respective source databases. B, The sets of EC numbers were filtered for reactions that produce CO_2_ in the respective models and were compared between all pairs of models. Jaccard index of 1 indicates equality of compared sets. The species were ordered according to their phylogeny based on multiple sequence alignment with neighbor joining of the RuBisCo large subunit (Katoh et al., 2019). The cladogram in B was generated using Phylo.io (Robinson et al., 2016).

Our update review indicates several directions for modeling of plant respiration (see Outstanding Questions). Efforts in the last decade have resulted in the assembly of high-quality metabolic networks for model plants and crops (for a comprehensive review, see (Tong et al., 2021)), providing the basis to address the noted challenges for modeling plant respiration (see “Challenges in modeling plant respiration” section). These models differ with respect to the considered pathways, namely, only from primary metabolism, expansion to pathways of secondary metabolism, and consideration of lipid metabolism (Correa et al., 2020)—all relevant when simulating and predicting the evolution of CO_2_ under different environments. They have been used in extracting context-specific metabolic networks by using transcriptomics, proteomics, and metabolomics data (Robaina Estévez and Nikoloski, 2014; Tong et al., 2021) and have been combined in multitissue models (Shaw and Cheung, 2020). Inspections indicated that these models (i) contain between 3.7% and 57.7% of all CO_2_-producing reactions present in plants (Fig. 5A), (ii) differ in the sets of considered CO_2_-producing reactions, with phylogenetically closer species showing higher similarity of these sets, and (iii) the models for maize (*Zea mays*) (Simons et al., 2014) and rice (*Oryza sativa spp. Indica*). Chatterjee et al. (2017) show the highest overall difference to all other models (Fig. 5B). For a comparison, the general characteristics of the compared models are provided in Supplemental Table S1.

**Figure 6:**
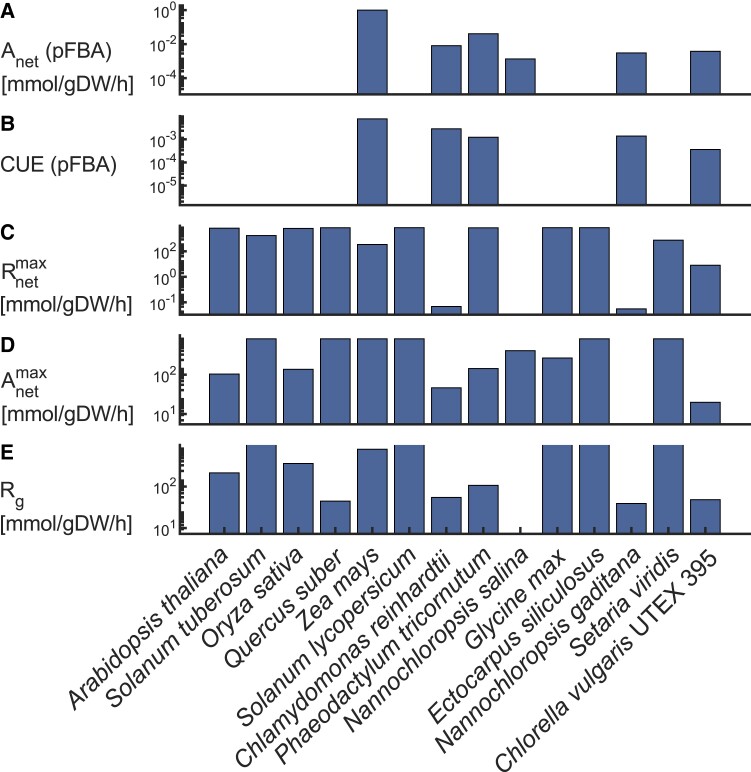
Functional analysis of CO_2_-related traits in genome-scale metabolic models of photosynthetic eukaryotes. The metabolic models, described in Figure 5, were filtered based on their ability to predict growth in flux balance analysis (FBA) using default constraints and objective function(s) included in the models. A, The difference in flux between CO_2_-consuming and producing reactions (*A*_*net*_) in parsimonious FBA. Here, values below 10^−9^ mmoL gDW^−1^ h^−1^ (gDW: gram dry weight) were not considered as they were below the solver feasibility tolerance. B, From the same flux distribution, we calculated the carbon use efficiency (CUE) by 1 − (0.5*v*_*o*_ + *v*_*r*_) /*v*_*c*_, where *v*_*r*_ = *v*_*c*_ − 0.5*v*_*o*_ − *A* (*v*_*c*_: flux though RuBisCO carboxylation reaction, *v*_*o*_: flux through RuBisCO oxygenation reaction, *v*_*r*_: estimated day respiration). C, Maximum CO_2_ release, assessed by maximization of an export reaction for CO_2_ ($R_{net}^{max}$). D, Maximum CO_2_ fixation, determined by maximizing the difference between CO_2_ uptake and CO_2_ export from the model ($A_{net}^{max})$. E, growth respiration (*R*_*g*_) as the sum of fluxes through all CO_2_-releasing reactions from the same flux distribution as in A and B. For more information on the calculations, see Supplemental Methods.

Functional analysis indicated that only half of the models yielded a net CO_2_ assimilation in the parsimonious flux balance analysis (pFBA) (Fig. 6A, Supplemental Information). For this analysis, we allowed the oxygenation to carboxylation ratio to range between 0.31 and 0.55, which corresponds to the ratio of intracellular partial pressure of O_2_ and CO_2_ (Farquhar et al., 1980) divided by the average specificity of RuBisCO for CO_2_ over O_2_ (see Supplemental Information). The values of net CO_2_ assimilation, *A*, were obtained by subtracting the sums of fluxes of all CO_2_-consuming reactions and all CO_2_-producing reactions (Fig. 6A). The fluxes through the carboxylation and oxygenation reactions of RuBisCO (*V*_*c*_, *V*_*o*_) were directly read out from the steady-state flux distributions obtained by pFBA, and the carbon use efficiency (CUE) was then calculated as follows: 1 − (0.5*V*_*o*_ + *R*_*d*_)/*V*_*c*_, following Eq. (2) (see Supplemental Information). Note, that CUE is defined by 1 − the ratio of net carbon gain to gross assimilation, and we used the expression 0.5*V*_*o*_ + *R*_*d*_ to estimate net carbon gain, and *V*_*c*_ is the estimate for gross assimilation. We found that the predicted carbon use efficiency ranged between 3.2 × 10^−4^ and 0.16 (Fig. 6B). To estimate the full capacity of CO_2_ release and fixation, we (1) maximized the flux through CO_2_ export and (2) maximized the difference between CO_2_ import and CO_2_ export at optimal growth as obtained from FBA (Fig. 6, C and D). The results indicated that many of the models are able to predict release of most, if not all the CO_2_ that was imported (Fig. 6C). However, all models are also able to predict net CO_2_ fixation to varying extents (Fig. 6D), demonstrating that fixation of CO_2_ into biomass can only be observed when explicitly included in the optimization; otherwise, half of the analyzed models will respire as much CO_2_ as they fix based on the predictions from pFBA. Finally, we estimated growth respiration, as the sum of fluxes of all CO_2_-producing reactions in our pFBA flux distribution (Fig. 6E). The predicted values ranged from 2 to 6419 mmoL gDW^−1^ h^−1^, where the green and brown algae species showed the lowest values. While the observed differences described earlier may in part be the result of varying biomass composition across the models, we calculated carbon molar fractions of the biomass reactions for some of the models, which did not show differences that relate to the results shown in Fig. 6 (Supplemental Fig. S3). However, differences over multiple orders of magnitude empathize the need of using measured photosynthesis and respiration rates to validate the predictions from these models. For instance, the predictions of photosynthesis using constraint-based models could largely be improved by consideration of estimated rates of CO_2_ uptake *V*_*c*_ or *V*_*o*_ from kinetic models.

The Arabidopsis core model (AraCore, Arnold and Nikoloski, 2014) is obtained in a bottom-up fashion, relying on the available literature and is restricted to primary metabolism, comprising 407 metabolites involved in 549 reactions. Due to its small size and high quality, we chose this model to showcase insights on modeling and modulating respiration. To investigate the relative contribution of reactions to the release of CO_2_, we ranked them based on the flux they carry, which was predicted by pFBA in a day and night-specific model, respectively (Arnold et al., 2015) (Supplemental Table S2). The relative growth rate was bounded by the optimal value, obtained from the day model, to allow for comparable results. Moreover, the resulting fluxes were scaled by the product of the predicted relative growth rate and the molar fraction of carbon in the biomass reaction to be able to quantify the amount of CO_2_ needed to produce one unit of carbon in biomass. As a result, the predictions showed that the scaled day respiration was higher than the scaled night respiration (307.56 > 262.32). The reaction that contributed most to CO_2_ release during the day was glycine dehydrogenase (EC 1.4.4.2), followed alanine synthase (EC 2.2.1.6/1.2.4.1) and chloroplastic isocitrate dehydrogenase (EC 1.1.1.42). With the night-specific model, we predicted the chloroplastic NADP-dependent 6PG dehydrogenase (EC 1.1.1.44) to be the contributing most to CO_2_ release, followed by PDH (EC 2.2.1.6/1.2.4.1) and the chloroplastic NADP-dependent malic enzyme (EC 1.1.1.40). We note that these results may differ substantially from those obtained from metabolic flux analysis (fitting labeling data to estimate fluxes) since the latter does not rely on the principle of parsimony, implying that reactions other than those presented in Supplemental Table S2 may contribute to net CO_2_ evolution.

The development of large-scale models of plant metabolism can be combined with advances in metabolic flux analysis to assess the contribution of different pathways and reactions to CO_2_ evolution. These advances include: (1) implementation of pipelines for automated generation of atom mappings for genome-scale metabolic models of plants (Huß et al., 2022) and (2) development of local approaches for flux estimation decrease computational challenges involved in flux estimation with large-scale models (Millard et al., 2020). However, the flux estimates depend strongly on the size (and detail) of the models considered, as recently reported for *Synechocystis* PCC 6,803 (Gopalakrishnan et al., 2018). For instance, for fluxes of reactions involved in respiration, this study found that the genome-scale model (with 683 reactions) estimated considerably lower fluxes compared to the core model (with 39 reactions). For the considered reactions, the maximum estimated flux from the genome-scale model was on average 38 times smaller than the maximum estimated using the core model, while the minimum flux found was on average of more than 10^7^ times smaller. Further, the relative ordering of fluxes (maximum, median) did not show correspondence between the estimations made from both models.

Access to flux distributions for a plant system (e.g. tissue or organ) provides the means to apply computational approaches for engineering of plant respiration using constraint-based modeling either at the reaction (Küken and Nikoloski, 2019) or the gene level (Razaghi-Moghadam and Nikoloski, 2021). Interestingly, the approaches for engineering are currently rooted in the growth-and-maintenance paradigm of respiration (Amthor et al., 2019), with little insights of how the manipulations can be achieved and what effects these manipulations will have in other parts of metabolism. In this regard, advances in constraint-based modeling that consider allocation or resources (Yang et al., 2018; Kerkhoven, 2022) allow the automated generation of engineering strategies targeted at the manipulation of allocations and simulating the effects of modifying the turnover of particular proteins in particular tissues or cell types.

We next assessed the effect of single and double knock-outs of reactions in the AraCore model on the sum of fluxes through CO_2_-producing reactions (*R*_*G*_). The resulting values were scaled by the product of carbon biomass fraction and predicted relative growth rate, as described earlier. Interestingly, we did not observe any combination of reaction knock-outs that led to a decrease in CO_2_ release per carbon in produced biomass (Supplemental Fig. S4). While the predicted growth rate decreases with decreasing *R*_*G*_, there are double knock-outs that substantially increase both the nominal and scaled *R*_*G*_. For instance, the simulated knock-outs for the mitochondrial complex 1 result in an 8.5-fold increase in the scaled *R*_*G*_ when paired with knock-outs of the peroxisomal O_2_ and water transport, glycolate transport between cytosol, peroxisome, and chloroplast, as well as peroxisomal glycolate oxidase and catalase. Moreover, the phosphglycerate (PGA) kinase decreased *R*_*G*_ in most knock-out pairs by reducing the predicted relative growth rate, while the scaled *R*_*G*_ is increased by at least 1.8-fold compared to the predictions for wild type. In addition, we asked whether there exist flux distributions that can result in a decrease in respiration. To this end, we minimized the ratio between the sum of fluxes through CO_2_-producing reactions and the product of relative growth rate and carbon molar fraction. We were able to find a flux distribution that reduced the ratio from 173.66 to 172.55. Although the decrease is small, it required substantial rerouting of fluxes involving complete deactivation and reactivation of reactions (Supplemental Table S3). These findings indicate that manipulation of plant respiration could be very challenging in practice.

The outlined perspectives are based on expanding the usage of constraint-based modeling and metabolic flux analysis to better understand steady-state plant respiration. However, by assuming the metabolic steady state, constrain-based modeling is not able to capture any time resolution or feedback effects that might play an important role in modeling plant respiration. While approaches have been developed to address these issues, this remains a large limitation of the constraint-based modeling framework (Mahadevan et al., 2002). Despite the advantage of being able to capture a genome-scale network of metabolism, the basic FBA principles lack detailed information on enzyme and metabolite concentrations as well as efficiency and affinity parameters of enzymes. Building kinetic models at this level necessitates improving the catalog and functional characterization of metabolite–protein interactions for enzymes involved in respiration (Reznik et al., 2017). Our survey of publicly available resources indicated that 65.7% and 45.6% of the considered EC numbers have reported inhibitors and activators in BRENDA (Chang et al., 2021). To assess which activating or inhibiting compounds are relevant for modeling metabolism, we matched the reported compounds with the AraCore model (Arnold and Nikoloski, 2014). As a result, 27 metabolites were found to act as an activator and 40 as inhibitors of reactions in respiratory pathways (Fig. 1) included in the model.

Finally, modeling developments have to go hand-in-hand with their usage in breeding lines with decreased respiration without negative impacts on growth. For instance, a recent study has shown that maize breeding has increased the number of florets per ear and ear growth rate, but not the vegetative shoot growth rate (Cagnola et al., 2021). This is attributed to an increase in the net CO_2_ exchange (of the ear leaf) largely accounted for by increased photosynthesis and by reduced respiration per unit leaf area, in line with reports in perennial ryegrass (*Lolium perenne* cv. S23) (Wilson, 1982). However, attempting to use respiration as a trait in breeding necessitates understanding its heritability and predictability by genetic markers alone (Tong and Nikoloski, 2021). By using an Arabidopsis diversity panel, night respiration was found to have low heritability (∼0.3), indicating that other approaches that account for environmental effects must be considered to improve its predictability (O’Leary et al., 2017). In this regard, it is important to survey the natural variability in the activity of key enzymes involved in plant respiration, as means for the identification of alleles that lead to decreased respiration. The latter can be addressed by (1) applying machine learning approaches to predict respiration from predictors that are easier to measure (e.g. hyperspectral reflectance characteristics) (Coast et al., 2019) and/or (2) employing approaches that couple constraint-based modeling with predictive models based on genetic markers, which are shown to improve prediction of metabolic phenotypes in Arabidopsis (Tong et al., 2020).

Ultimately, such advances in understanding respiration at the level of metabolism will have to be coupled with mechanistic modeling of temperature effects on metabolism and physiology and models that account for leaf protein as well as amount and rate of phloem loading per unit leaf area. This modeling paradigm may help in understanding what are the factors that contribute to acclimation of plant respiration to increased temperature by using hybrid, mechanistic modeling frameworks. Such research efforts will provide stepchange in incorporation of respiration in terrestrial biosphere models and earth system models, beyond the existing paradigms.

Outstanding questions Box

- Can we develop enzyme-constrained and resource allocation models for plants to facilitate modeling the costs of protein allocation, translation, and transcription that contribute to respiration in the context of predicting plant growth?
- Can we generate organ-specific metabolic models to facilitate the mechanistic study of differences in respiration between plant organs?
- How can we obtain insights in the difficulty to modulate day and night respiration by applying computational approach for design of metabolic engineering strategies from the constraint-based modeling framework and apply these insights in crop breeding for decreased respiration?
- How can we integrate predictions about respiration from mechanistic metabolic models into terrestrial biosphere models and earth system models to gain insights in the acclimation of respiration and its response to future climate scenarios?

## Supplementary Material

kiad054_Supplementary_Data

## Supplemental data

The following materials are available in the online version of this article.

## Supplemental Methods

**Supplemental Figure S1**. Flux of CO_2_-producing reactions in the AraCore day- and night-specific models.

**Supplemental Figure S2**. Gene expression of respiratory enzymes across different plant tissues.

**Supplemental Figure S3**. Carbon molar fraction in selected models of photosynthetic eukaryotes.

**Supplemental Figure S4**. Changes in nominal and scaled growth respiration in single- and double-reaction knock-outs.

**Supplemental Table S1**. General information on models analyzed in this study.

**Supplemental Table S2**. Ranking of CO_2_-producing reactions for the day- and night-specific AraCore adaptations.

**Supplemental Table S3**. Activated and deactivated reactions in wild-type (day) pFBA flux distribution and flux distribution obtained upon minimization of the ratio between CO_2_ release and growth rate times molar fraction of carbon in biomass.
