# Supplementary material for: Toward mechanistic modeling and rational engineering of plant respiration
Source: Plant Physiol. 2023 Jan 31;191(4):2150–66. doi: 10.1093/plphys/kiad054 (PMC10069892; doi:10.1093/plphys/kiad054)
Supplement: kiad054_Supplementary_Data [file kiad054_supplementary_data.pdf]

Supplementary Information for

Towards mechanistic modelling and rational engineering of plant respiration

Philipp Wendering and Zoran Nikoloski

Systems Biology and Mathematical Modeling, Max Planck Institute of Molecular Plant Physiology, 14476 Potsdam, Germany

Bioinformatics, Institute of Biochemistry and Biology, University of Potsdam, 14476 Potsdam, Germany

Supplemental Methods

Modelling CO_2 assimilation and respiration in genome-scale metabolic models

To obtain a physiologically meaningful flux distribution for a photoautotrophic growth, the ratio of fluxes for the oxygenation to carboxylation reactions of RuBisCO (V_o, V_c) must be constrained; this is needed to avoid zero flux through photorespiration (except for organisms with carbon concentrating mechanisms, where photorespiration is negligible). Since we attempted to get an overview of photosynthetic and respiratory capabilities based on genome-scale metabolic models of 15 species, we derived a single ratio for V_o and V_c that represents the average over many measurements. To this end, we first obtained values for the specificity of RuBisCO for CO_2 over O_2, $S_{c/o}$, from three different studies (Parry et al., 1989; Zhu et al., 1992; Hermida-Carrera et al., 2016). These values were converted from (unitless) mol/mol to (unitless) bar/bar by using a factor determined from the results of Walker and colleagues (Walker et al., 2013), where $S_{c/o}$ was given in mol/mol and bar/bar. Finally, the value for the oxygenation to carboxylation ratio, ϕ, was calculated using intercellular partial pressures for CO_2 (C) and O_2 (O) given in Farquhar et al. (1980) (Farquhar et al., 1980):

$$\phi = \frac{1}{S_{c/o}} \frac{O}{C}.$$

In the optimization programs, the ratio between v_o and v_c (correspond to V_o and V_c) was fixed within two standard deviations (σ) of the distribution of ϕ values:

$$\phi - 2\sigma \leq \frac{v_o}{v_c} \leq \phi + 2\sigma.$$

The average value for ϕ was 0.43 and σ was 0.06, so the ratio of oxygenation to carboxylation flux was allowed to range between 0.31 and 0.55.

First, we performed flux balance analysis (FBA) with additional constrains on the ratio of V_o and V_c and contraints from loopless FBA (Schellenberger et al., 2011). These additional constraints were applied in three optimization problems, which essentially differed in their objectives. For all simulations, the reversible reactions in the models were split into two irreversible reactions.

I Maximization of biomass precursor production

$$\max v_{bio}$$

s.t.

$$\mathbf{Sv} = \mathbf{0}$$

$$\mathbf{v^{min}} \leq \mathbf{v} \leq \mathbf{v^{max}}$$

$$-1000(1 - a_i) \leq v_i \leq 1000a_i$$

$$-1000a_i + 1(1 - a_i) \leq G_i \leq -1a_i + 1000(1 - a_i)$$

$$\mathbf{S_{int}G} = \mathbf{0}$$

$$a \in \{0,1\}$$

$$G_i \in \mathbb{R}$$

$$i \in internal$$

$\mathbf{S}$ is the stoichiometric matrix, $\mathbf{S_{int}}$ is the part of $\mathbf{S}$ that only contains internal reactions i.e., excludes import/export reactions, and $\mathbf{v}$ denotes the vector of fluxes through all reactions in the model. The binary variable a_i indicated for each internal reaction i , whether its direction is forward or backward. The vector $\mathbf{G}$ contains the Gibbs free energy proxies of the reactions (Schellenberger et al., 2011).

In models that do not contain a single biomass reaction, rather than maximizing v_{bio}, we maximize the sum of fluxes of reactions that export biomass components.

II Maximization of CO_2 release

To obtain the maximum possible CO_2 release, we maximized the flux through an artificial reaction that draws CO_2 from the system, while guaranteeing for at least 99% of the optimal objective value determined in problem I.

$$\max v_{CO_2\,sink}$$

s.t.

constraints from problem I

$$\sum v_{bio} = 0.99 \sum v_{bio}^{*},$$

where $\sum v_{bio}^{*}$ is the optimal value of problem I. The variable $v_{CO_2\,sink}$ denotes a reaction that exports CO_2 from the system.

III Maximization of CO_2 assimilation

To estimate the maximum possible CO_2 assimilation, all previously existing CO_2 uptake or release reactions were blocked and the difference between an artificial CO_2 uptake and release reaction was maximized. Again, the sum of biomass reaction(s) was fixed to 99% of the optimal value (problem I).

$$\max v_{CO_2\,uptake} - v_{CO_2\,sink}$$

s.t.

constraints from problem I

$$\sum v_{bio} = 0.99 \sum v_{bio}^{*}.$$

Here, $v_{CO_2\,uptake}$ represents a reaction that produces CO_2 and thus imports it into the system.

All three optimizations were followed by minimization of the first nom of the flux distribution (i.e. the sum of the absolute values of fluxes). The simulations were carried out using the COBRA toolbox function *optimizeCbModel* with options corresponding to the additional constraints mentioned above. All optimization problems were solved using the Gurobi solver (Gurobi Optimization, 2021) with a feasibility tolerance of 10^{-9}.

Calculation of net CO2 assimilation and carbon use efficiency from day models from predicted flux distributions

The values for net CO_2 assimilation (A) and carbon use efficiency were obtained from the solution to problem I. Net assimilation of CO_2 was determined by subtracting the sums of fluxes of all CO_2-consuming reactions from all CO_2-producing reactions. Carbon use efficiency (CUE) was calculated by

$$CUE = 1 - \frac{0.5v_o + R_d}{v_c}.$$

In this formula, R_d corresponds to day respiration, which was determined by

$$R_d = v_c - 0.5v_o - A.$$

We note that this definition differs from the classical, given by $CUE = 1 - \frac{\text{net carbon gain}}{\text{gross carbon assimilation}}$, that involves the net CO_2 assimilation, day respiration, and night respiration over a period of time. From the specification, it is expected that over a day, the two formulations result in proportional values.

Calculation of the carbon molar fraction in the biomass reaction

First, the mass fraction of carbon atoms ($MF_{C,i}$) for a metabolite (substrate) i in the biomass reaction was calculated by:

$$MF_{C,i} = \frac{MW_i}{n_{C,i} \cdot MW_C},$$

where MW_i denotes the molecular weight of the metabolite, $n_{C,i}$ represents the number of carbon atoms in the sum formula of i, and MW_C is the molecular weight of carbon.

Second, the weight of each metabolite, w_i, was calculated by:

$$w_i = S_{i,bio} \cdot MW_i,$$

where $S_{i,bio}$ denotes the stoichiometric coefficient of metabolite i in the biomass reaction with unit mmol per gram dry weight (gDW).

Next, the weight of carbon per metabolite weight ($w_{C,i}$) was calculated using the mass fractions of carbon atoms calculated above:

$$w_{C,i} = w_i \cdot MF_{C,i}.$$

Finally, the molar fraction of carbon in the biomass reaction was determined by dividing the sum of carbon weights, divided by the molecular weight of carbon ($\lambda \left[\frac{mmol}{gDW}\right]$):

$$\lambda = 1000 \cdot \frac{w_{C,i}}{MW_C}.$$

Day- and night-specific modelling of respiration

The AraCore model (Arnold and Nikoloski, 2014) was updated by integrating changes in reaction lower and upper bounds (Arnold et al., 2015). Moreover, the ratio between carboxylation flux and oxygenation flux was constrained for the day-specific model as described above in the beginning of the document. To enable flux through the biomass reaction in the night-specific model, we made the following that differ from the changes in the cited publication.

Reaction ID	Reaction equation	Lower Bound	Upper bound	Comment
Ex_starch5[h]	starch5[h] <=>	-1000	1000	Added a reaction to enable uptake of starch
Ex_Gly_h	Gly[h] <=>	-1000	1000	Changed bounds to enable uptake of glycine
Ex_Glc	Glc[c] ->	0	1000	Changed bounds to disable uptake of glucose

Flux distributions for both models were obtained by solving *problem I*, followed by minimization of the sum of all fluxes. In the night-specific model, the predicted growth rate was limited to the optimal value for the relative growth rate of the day-specific model. The values for growth respiration (R_G) were obtained by summing up all predicted flux values of CO_2-producing reactions, except for transport and import reactions ("Tr_*" and "Im_*").

The obtained values for R_G were then scaled to the product of the predicted relative growth rate (μ) and the molar fraction of carbon in the biomass reaction (λ), as described above:

$$R'_G = \frac{R_G}{\mu \cdot \lambda}.$$

Modelling of single- and double knock-outs and their effect on respiration

To this ends, the AraCore model was used with constraints on the lower and upper bound of the ratio between fluxes through oxygenation and carboxylation reactions. Single and double knock-outs were simulated by blocking the individual reactions of pairs of reactions. Flux distributions were predicted by solving *problem I*. The predicted relative growth rates and R_G values were then used to calculate the value for R'_G, as described above.

Minimization of growth respiration

The AraCore model was used with constraints on the ratio between oxygenation and carboxylation fluxes as described above. All reversible reactions in the model were split into two irreversible reactions. The following linear fractional program was then solved, which minimizes the ration between fluxes through CO_2-producing reactions ($v^+_{CO_2}$) and the product of μ and λ (using Charnes-Cooper transformation):

Original optimization program:

$$\min \frac{\sum v_{CO_2}^{+}}{\mu \cdot \lambda}$$

s.t.

$$Sv = 0$$

$$0 \leq v \leq v^{max}$$

Charnes-Cooper transformation:

$x = \frac{1}{\lambda \cdot \mu} v, t = \frac{1}{\lambda \cdot \mu}$

Transformed optimization program:

$$\min \sum x_{CO_2}^{+}$$

s.t.

$$\boldsymbol{Sx = 0}$$

$$\boldsymbol{0 \leq x \leq v^{max} t}$$

$$\lambda x_{bio} = 1$$

$$t \geq 0$$

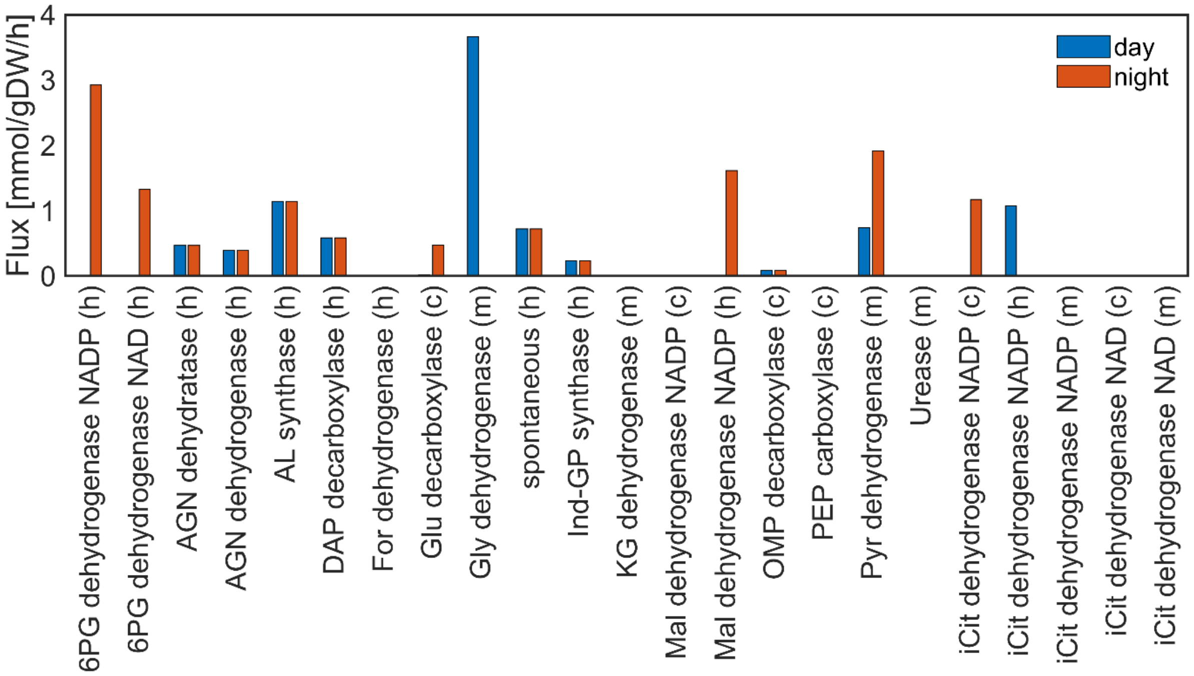

Supplemental Figure S1. Flux of CO_2-producing reactions in the AraCore day- and night-specific models. The AraCore model was updated to a day- and night- specific model (Arnold and Nikoloski, 2014; Arnold et al., 2015; see **Supplemental Methods** for more detailed information). The relative growth rate for both simulations was kept equal to the optimal value predicted using the day-specific model. Flux distributions were predicted using parsimonious flux balance analysis. The flux through CO_2-producing reactions is shown which are contained in both the day- and night-specific model, (**Fig. 1** for graphical representation). The abbreviations c, m, and h indicate the compartments, where the reactions are located (c: cytosol, m: mitochondrion, h: chloroplast). gDW denotes gram dry weight.

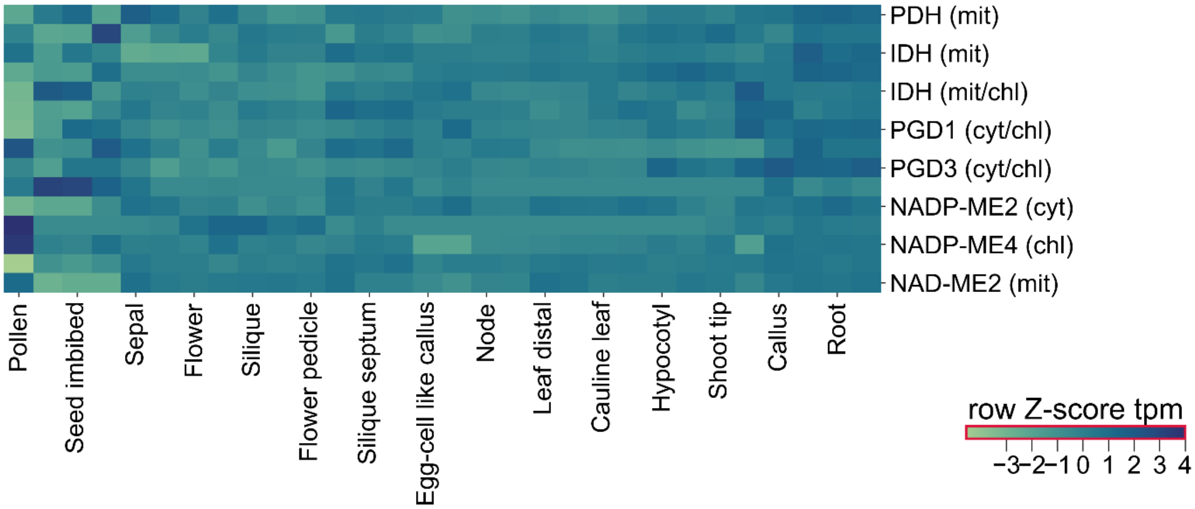

Supplemental Figure S2. Gene expression of respiratory enzymes across different plant tissues. Transcript abundances across 30 tissues were obtained for the enzymes described in **Fig. 1** (Mergner et al., 2020)**.** The tpm (transcript per million) values across the different tissues were Z-transformed for each enzyme separately. For enzyme complexes, the minimum value of the subunit expression values is shown. tpm values are calculated by multiplying the normalized read counts (number of reads mapped to transcript / length of transcript) by 10^6. PDH: pyruvate dehydrogenase, IDH isocitrate dehydrogenase, 2OGDH: 2-oxoglutarate dehydrogenase, PGD: phosphogluconate dehydrogenase, ME: malic enzyme. The abbreviations cyt, mit, per, and chl indicate the compartments, where the reactions are located (cyt: cytosol, mit: mitochondrion, per: peroxisom, chl: chloroplast).

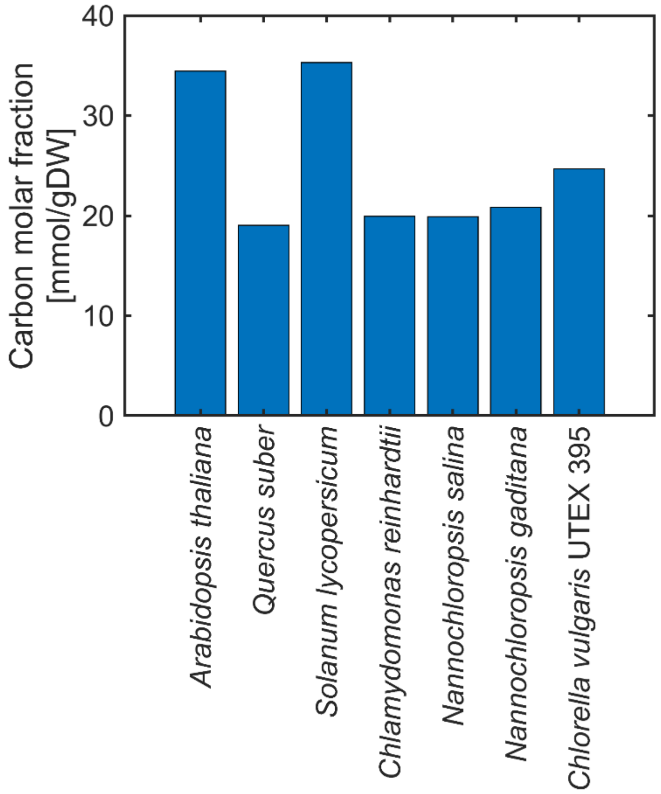

Supplemental Figure S3. Carbon molar fraction in selected models of photosynthetic eukaryotes. The molar fraction of carbon was calculated as described in the **Supplementary Methods**. The values were scaled by the sum of metabolite weights that are substrates in the biomass reaction to make the value comparable between models. This value should theoretically be 1 g/gDW but this is not the case for all models. The calculation was performed on all models shown in **Fig. 5**, but here only we only those models are represented for which the calculation of the carbon molar fraction could be performed. gDW denotes gram dry weight.

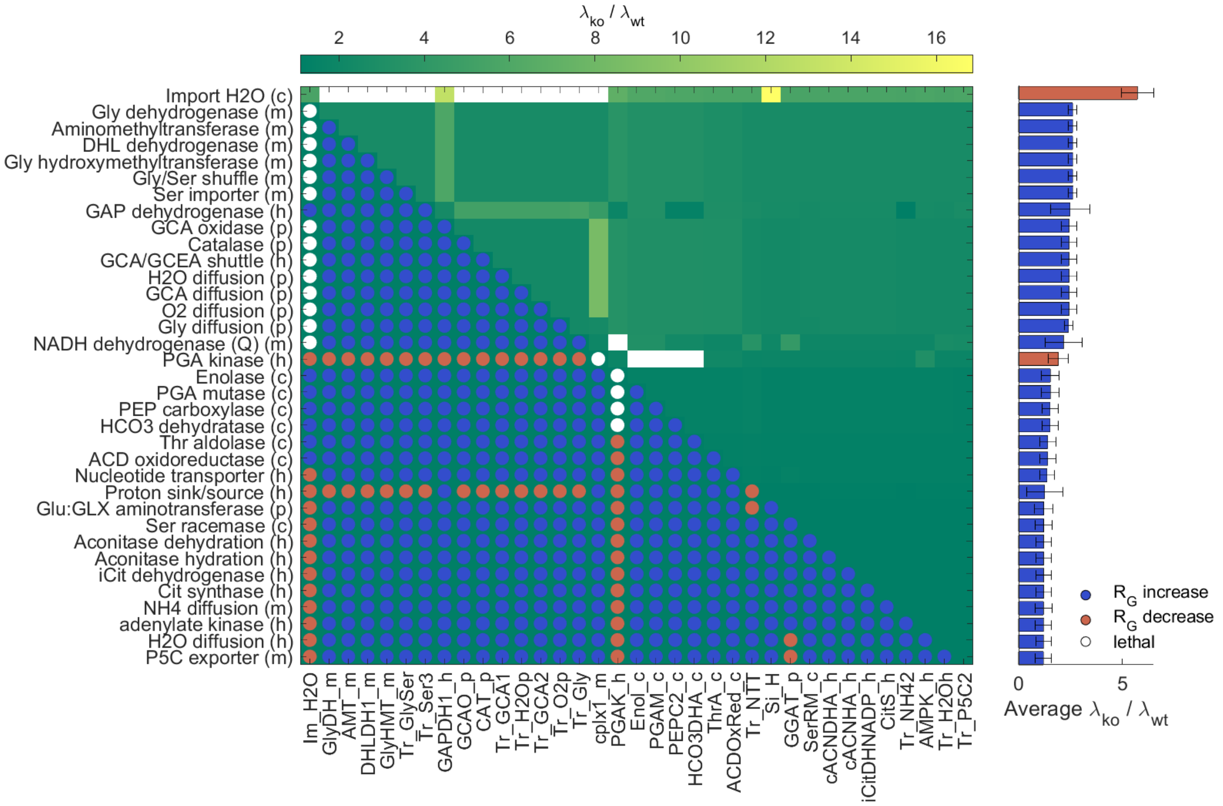

Supplemental Figure S4. Changes in nominal and scaled growth respiration in single and double reaction knock-outs. We predicted flux distributions of all possible single and double knock-outs of reactions in the AraCore model (Arnold and Nikoloski, 2014). The sum of fluxes through CO_2-producing reactions was used to approximate nominal growth respiration, R_G. The values for R_G were classified as lower (orange) or higher (blue) compared to nominal R_G of the wild-type model (lower left triangle). The upper right triangle shows the ratio of scaled growth respiration, λ, between the simulated knock-out and the wild-type model. The values for λ were obtained by dividing the nominal R_G by the product of predicted relative growth rate and the molar fraction of carbon in the biomass reaction. White color indicates predicted lethal knock-outs. The heatmap only shows the top ten percent of reactions with respect to their average change in $\lambda_{ko}/\lambda_{wt}$. The average $\lambda_{ko}/\lambda_{wt}$ values per reaction across all combinations (n=549) is shown on the right with the same colour coding (i.e. lower (orange) or higher (blue) compared to nominal R_G of the wild-type model). Error bars denote standard deviation. The abbreviations c, m, p, and h indicate the compartments, where the reactions are located (c: cytosol, m: mitochondrion, p: peroxisome, h: chloroplast). The underlying modelling is explained in the **Supplemental Methods**.

Supplemental Table S1. General information on models analysed in this study.

AuthorYear	Species	Number of genes	reactions	metabolites	compartments
ArnoldNikoloski2014	*Arabidopsis thaliana*	634	549	407	6
Botero2018	*Solanum tuberosum*	2751	2064	1940	3
Chatterjee2017	*Oryza sativa*	3602	1136	1330	4
Cunha2022	*Quercus suber*	7871	6938	6481	10
DalMolin2010	*Saccharum officinarum*	3881	1243	1420	12
DalMolin2010	*Sorghum bicolor*	3557	1243	1420	12
Simons2014	*Zea mays*	6535	8525	9025	17
Gerlin2022	*Solanum lycopersicum*	3433	2183	2019	5
Hay2014	*Brassica napus*	962	669	672	11
Imam2015	*Chlamydomonas reinhardtii*	1460	2394	1845	11
Levering2016	*Phaeodactylum tricornutum*	1025	2156	1704	6
Klanchui2018	*Arthropira plantensis* C1	886	1096	994	7
Loira2017	*Nannochloropsis salina*	935	2345	1985	10
Moreira2019	*Glycine max*	6127	3001	2814	5
Pfau2018	*Medicago truncatula*	3403	2909	2780	10
Prigent2014	*Ectocarpus siliculosus*	NaN	1866	2020	1
SarkarMaranas2020	*Populus trichocarpa*	7188	9440	9703	9
Shah2017	*Nannochloropsis gaditana*	1321	1918	1862	4
ShawCheung2019	*Setaria viridis*	3376	2473	2429	5
Zuniga2016	*Chlorella vulgaris* UTEX 395	843	2294	1770	6

Supplemental Table S2. Ranking of CO_2-producing reactions for the day- and night-specific AraCore adaptations. The column "Flux in pFBA" shows fluxes obtained from solving *problem I* (see Supplementary Methods) followed by minimization of the sum of fluxes. The last column contains the same fluxes, scaled by the product of the predicted relative growth rate and the carbon molar fraction in the biomass reaction. The abbreviations c, m, p, and h indicate the compartments, where the reactions are located (c: cytosol, m: mitochondrion, p: peroxisome, h: chloroplast). gDW denotes gram dry weight.

Reaction ID	Reaction name	EC Numbers	Reaction subsystem / pathway	Reaction formula	Flux in pFBA [mmol/gDW/h]	Scaled pFBA flux
**Day**						
Tr_CO2h_f	CO2 diffusion	--	transport	CO2[c] -> CO2[h]	74.36	1100.34
Im_CO2_f	Import CO2	--	import	-> CO2[c]	67.58	1000.00
Tr_CO2m_b	CO2 diffusion	--	transport	CO2[m] -> CO2[c]	13.98	206.93
GlyDH_m	Gly dehydrogenase	1.4.4.2	photorespiration	H[m] + Gly[m] + LPL[m] -> CO2[m] + amDHP[m]	13.44	198.81
ALS1_h	AL synthase	2.2.1.6/ 1.2.4.1	isoleucine synthesis, leucine synthesis, valine synthesis	H[h] + Pyr[h] + ThPP[h] -> CO2[h] + H-Eth-ThPP[h]	1.29	19.15
iCitDHNADP_h	iCit dehydrogenase	1.1.1.42	pyruvate metabolism	NADP[h] + iCit[h] -> NADPH[h] + CO2[h] + KG[h]	1.15	17.07
PyrDH1_m	Pyr dehydrogenase	2.2.1.6/ 1.2.4.1	pyruvate decarboxylation	Pyr[m] + ThPP[m] + H[m] -> H-Eth-ThPP[m] + CO2[m]	0.55	8.12
IPODC_h	spontaneous	--	leucine synthesis	H[h] + IPO[h] -> CO2[h] + 4MOP[h]	0.52	7.70
DAPDC_h	DAP decarboxylase	4.1.1.20	lysine synthesis	H[h] + mDAP[h] -> CO2[h] + Lys[h]	0.34	5.02
AGNDA_h	AGN dehydratase	4.2.1.91	phenylalanine synthesis	H[h] + AGN[h] -> H2O[h] + CO2[h] + Phe[h]	0.22	3.23
AGNDH_h	AGN dehydrogenase	1.3.1.78	tyrosine synthesis	NADP[h] + AGN[h] -> NADPH[h] + CO2[h] + Tyr[h]	0.15	2.28
IndGPS_h	Ind-GP synthase	4.1.1.48	tryptophan synthesis	H[h] + CPD-Ru5P[h] -> H2O[h] + CO2[h] + Ind-GP[h]	0.06	0.82
OMPDC_c	OMP decarboxylase	4.1.1.23	UMP synthesis	H[c] + OMP[c] -> CO2[c] + UMP[c]	0.01	0.11
GluDC_c	Glu decarboxylase	4.1.1.15	glutamate degradation	H[c] + Glu[c] -> CO2[c] + GABA[c]	3.08E-04	4.56E-03
iCitDHNADP_c	iCit dehydrogenase	1.1.1.42	pyruvate metabolism	H[c] + NADP[c] + iCit[c] -> CO2[c] + NADPH[c] + KG[c]	2.33E-08	3.45E-07
PEPC1_c	PEP carboxylase	4.1.1.49	gluconeogenesis	ATP[c] + OAA[c] -> ADP[c] + PEP[c] + CO2[c]	0.00	0.00

iCitDHNAD_m	iCit dehydrogenase	1.1.1.41	tricarboxylic acid cycle	NAD[m] + iCit[m] -> CO2[m] + NADH[m] + KG[m]	0.00	0.00
KGDH_m	KG dehydrogenase	1.2.4.2	tricarboxylic acid cycle	H[m] + LPA[m] + KG[m] -> CO2[m] + S-DHL[m]	0.00	0.00
6PGDHNAD_h_f	6PG dehydrogenase	1.1.1.44	pentose phosphate pathway	NAD[h] + 6PG[h] -> CO2[h] + Ru5P[h] + NADH[h]	0.00	0.00
6PGDHNADP_h_f	6PG dehydrogenase	1.1.1.44	pentose phosphate pathway	NADP[h] + 6PG[h] -> NADPH[h] + CO2[h] + Ru5P[h]	0.00	0.00
MalDH2NADP_c	Mal dehydrogenase	1.1.1.40	pyruvate metabolism	H[c] + Mal[c] + NADP[c] -> CO2[c] + NADPH[c] + Pyr[c]	0.00	0.00
MalDH2NADP_h	Mal dehydrogenase	1.1.1.40	pyruvate metabolism	NADP[h] + Mal[h] -> NADPH[h] + CO2[h] + Pyr[h]	0.00	0.00
iCitDHNAD_c	iCit dehydrogenase	1.1.1.41	pyruvate metabolism	H[c] + NAD[c] + iCit[c] -> NADH[c] + CO2[c] + KG[c]	0.00	0.00
iCitDHNADP_m	iCit dehydrogenase	1.1.1.42	pyruvate metabolism	iCit[m] + NADP[m] -> CO2[m] + KG[m] + NADPH[m]	0.00	0.00
Urease_m	Urease	3.5.1.5	proline synthesis	H[m] + H2O[m] + urea[m] -> CO2[m] + 2 NH4[m]	0.00	0.00
Tr_CO2m_f	CO2 diffusion	--	transport	CO2[c] -> CO2[m]	0.00	0.00
ForDH_h_b	For dehydrogenase	1.2.1.2	THF recycling	NAD[h] + For[h] -> CO2[h] + NADH[h]	0.00	0.00
Tr_CO2h_b	CO2 diffusion	--	transport	CO2[h] -> CO2[c]	0.00	0.00
**Night**						
Tr_CO2h_b	CO2 diffusion	--	transport	CO2[h] -> CO2[c]	12.56	185.86
6PGDHNADP_h_f	6PG dehydrogenase	1.1.1.44	pentose phosphate pathway	NADP[h] + 6PG[h] -> NADPH[h] + CO2[h] + Ru5P[h]	8.55	126.57
PyrDH1_m	Pyr dehydrogenase	2.2.1.6/ 1.2.4.1	pyruvate decarboxylation	Pyr[m] + ThPP[m] + H[m] -> H-Eth-ThPP[m] + CO2[m]	3.67	54.30
Tr_CO2m_b	CO2 diffusion	--	transport	CO2[m] -> CO2[c]	3.67	54.30
MalDH2NADP_h	Mal dehydrogenase	1.1.1.40	pyruvate metabolism	NADP[h] + Mal[h] -> NADPH[h] + CO2[h] + Pyr[h]	2.61	38.66
6PGDHNAD_h_f	6PG dehydrogenase	1.1.1.44	pentose phosphate pathway	NAD[h] + 6PG[h] -> CO2[h] + Ru5P[h] + NADH[h]	1.76	26.02
iCitDHNADP_c	iCit dehydrogenase	1.1.1.42	pyruvate metabolism	H[c] + NADP[c] + iCit[c] -> CO2[c] + NADPH[c] + KG[c]	1.38	20.38
ALS1_h	AL synthase	2.2.1.6/ 1.2.4.1	isoleucine synthesis, leucine synthesis, valine synthesis	H[h] + Pyr[h] + ThPP[h] -> CO2[h] + H-Eth-ThPP[h]	1.29	19.15
IPODC_h	spontaneous	--	leucine synthesis	H[h] + IPO[h] -> CO2[h] + 4MOP[h]	0.52	7.70

DAPDC_h	DAP decarboxylase	4.1.1.20	lysine synthesis	H[h] + mDAP[h] -> CO2[h] + Lys[h]	0.34	5.02
GluDC_c	Glu decarboxylase	4.1.1.15	glutamate degradation	H[c] + Glu[c] -> CO2[c] + GABA[c]	0.22	3.31
AGNDA_h	AGN dehydratase	4.2.1.91	phenylalanine synthesis	H[h] + AGN[h] -> H2O[h] + CO2[h] + Phe[h]	0.22	3.23
AGNDH_h	AGN dehydrogenase	1.3.1.78	tyrosine synthesis	NADP[h] + AGN[h] -> NADPH[h] + CO2[h] + Tyr[h]	0.15	2.28
IndGPS_h	Ind-GP synthase	4.1.1.48	tryptophan synthesis	H[h] + CPD-Ru5P[h] -> H2O[h] + CO2[h] + Ind-GP[h]	0.06	0.82
OMPDC_c	OMP decarboxylase	4.1.1.23	UMP synthesis	H[c] + OMP[c] -> CO2[c] + UMP[c]	0.01	0.11
PEPC1_c	PEP carboxylase	4.1.1.49	gluconeogenesis	ATP[c] + OAA[c] -> ADP[c] + PEP[c] + CO2[c]	0.00	0.00
iCitDHNAD_m	iCit dehydrogenase	1.1.1.41	tricarboxylic acid cycle	NAD[m] + iCit[m] -> CO2[m] + NADH[m] + KG[m]	0.00	0.00
KGDH_m	KG dehydrogenase	1.2.4.2	tricarboxylic acid cycle	H[m] + LPA[m] + KG[m] -> CO2[m] + S-DHL[m]	0.00	0.00
GlyDH_m	Gly dehydrogenase	1.4.4.2	photorespiration	H[m] + Gly[m] + LPL[m] -> CO2[m] + amDHP[m]	0.00	0.00
MalDH2NADP_c	Mal dehydrogenase	1.1.1.40	pyruvate metabolism	H[c] + Mal[c] + NADP[c] -> CO2[c] + NADPH[c] + Pyr[c]	0.00	0.00
iCitDHNAD_c	iCit dehydrogenase	1.1.1.41	pyruvate metabolism	H[c] + NAD[c] + iCit[c] -> NADH[c] + CO2[c] + KG[c]	0.00	0.00
iCitDHNADP_h	iCit dehydrogenase	1.1.1.42	pyruvate metabolism	NADP[h] + iCit[h] -> NADPH[h] + CO2[h] + KG[h]	0.00	0.00
iCitDHNADP_m	iCit dehydrogenase	1.1.1.42	pyruvate metabolism	iCit[m] + NADP[m] -> CO2[m] + KG[m] + NADPH[m]	0.00	0.00
Urease_m	Urease	3.5.1.5	proline synthesis	H[m] + H2O[m] + urea[m] -> CO2[m] + 2 NH4[m]	0.00	0.00
Tr_CO2h_f	CO2 diffusion	--	transport	CO2[c] -> CO2[h]	0.00	0.00
Tr_CO2m_f	CO2 diffusion	--	transport	CO2[c] -> CO2[m]	0.00	0.00
ForDH_h_b	For dehydrogenase	1.2.1.2	THF recycling	NAD[h] + For[h] -> CO2[h] + NADH[h]	0.00	0.00

Supplemental Table S3. Activated and deactivated reactions in wild type (day) pFBA flux distribution and flux distribution obtained upon minimization of the ratio between CO_2 release and growth rate times molar fraction of carbon in biomass. The abbreviations c, m, p, and h indicate the compartments, where the reactions are located (c: cytosol, m: mitochondrion, p: peroxisome, h: chloroplast). gDW denotes gram dry weight.

		**Flux [mmol/gDW/h]**	
**Reaction ID**	**Reaction name**	**WT**	**minimization**
MalDH3_m	Mal dehydrogenase	19.16	0.00
GABATA1_m_f	GABA transaminase	0.00	0.22
SCASeADH_m	SCA-SeA dehydrogenase	0.00	0.22
Tr_DTC3C_f	Di-/Tri-carboxylate carrier	18.94	0.00
Tr_BAT15_f	B0,+-type amino acid transporter 1	0.00	0.22
Tr_Glu1_f	Glu diffusion	2.18	0.00
MalDH1_c_b	Mal dehydrogenase	0.00	3.26
MalDH1_m_b	Mal dehydrogenase	0.00	16.61
Tr_DTC1B_b	Di-/Tri-carboxylate carrier	0.00	0.17
Tr_DTC1C_b	Di-/Tri-carboxylate carrier	0.39	0.00
Tr_DTC2B_b	Di-/Tri-carboxylate carrier	0.00	16.44
Tr_KG_b	KG diffusion	2.18	0.00

Supplemental References

Arnold A, Nikoloski Z (2014) Bottom-up Metabolic Reconstruction of Arabidopsis and Its Application to Determining the Metabolic Costs of Enzyme Production. Plant Physiol **165**: 1380–1391

Arnold A, Sajitz-Hermstein M, Nikoloski Z (2015) Effects of Varying Nitrogen Sources on Amino Acid Synthesis Costs in *Arabidopsis thaliana* under Different Light and Carbon-Source Conditions. PLoS One **10**: e0116536

Farquhar GD, von Caemmerer S, Berry JA (1980) A biochemical model of photosynthetic CO_2 assimilation in leaves of C_3 species. Planta **149**: 78–90

Gurobi Optimization L (2021) Gurobi Optimizer Reference Manual (https://www.gurobi.com).

Hermida-Carrera C, Kapralov M V., Galmés J (2016) Rubisco Catalytic Properties and Temperature Response in Crops. Plant Physiol **171**: 2549–2561

Mergner J, Frejno M, List M, Papacek M, Chen X, Chaudhary A, Samaras P, Richter S, Shikata H, Messerer M, et al (2020) Mass-spectrometry-based draft of the Arabidopsis proteome. Nature **579**: 409–414

Parry MAJ, Keys AJ, Gutteridge S (1989) Variation in the specificity factor of C_3 higher plant rubiscos determined by the total consumption of ribulose-P_2. J Exp Bot **40**: 317–320

Schellenberger J, Lewis NE, Palsson B (2011) Elimination of thermodynamically infeasible loops in steady-state metabolic models. Biophys J **100**: 544–553

Walker B, Ariza LS, Kaines S, Badger MR, Cousins AB (2013) Temperature response of in vivo Rubisco kinetics and mesophyll conductance in *Arabidopsis thaliana*: Comparisons to *Nicotiana tabacum*. Plant, Cell Environ **36**: 2108–2119

Zhu G, Jensen RG, Hallick RB, Wildner GF (1992) Simple Determination of the CO_2 /O_2 Specificity of Ribulose-1,5-Bisphosphate Carboxylase/Oxygenase by the Specific Radioactivity of [^{14}C]Glycerate 3-Phosphate. Plant Physiol **98**: 764–768
